# Smartphone Use Time and Total Screen Time Among Students Aged 10–19 and the Effects on Academic Stress: A Large Longitudinal Cohort Study in Shanghai, China

**DOI:** 10.3389/fpubh.2022.869218

**Published:** 2022-05-17

**Authors:** Shaojie Liu, Yukun Lan, Bo Chen, Gengsheng He, Yingnan Jia

**Affiliations:** Key Lab of Public Health Safety of the Ministry of Education, School of Public Health, Fudan University, Shanghai, China

**Keywords:** academic stress, smartphone use time, screen time, cohort study, adolescent health

## Abstract

**Objective:**

This study aimed to assess smartphone use time and total screen time among students aged 10–19 in Shanghai, China, and examine their effects on academic stress.

**Methods:**

Baseline and follow-up surveys were conducted in December 2017 and December 2018, respectively, using a cohort study design with 1,771 participants. Questionnaire surveys and physical examinations of participants were conducted by trained investigators and professional school physicians, respectively. The self-administered questionnaire mainly covered demographic information, academic stress, smartphone use time, total screen time, and other lifestyle behaviors.

**Results:**

The average smartphone use time of primary, middle, and high school students was 0.76 ± 0.90, 1.34 ± 1.29, and 2.39 ± 1.66 h/day, respectively; total screen time was 2.60 ± 2.63, 2.65 ± 3.39, and 3.52 ± 2.7 h/day, respectively (*P* < 0.001). The academic stress scores of primary, middle, and high school students were 9.25 ± 3.96, 11.97 ± 4.58, and 15.06 ± 5.10 (out of 30), respectively. The smartphone use time and total screen time were positively associated with academic stress score, with β values of 0.307 (95% CI: 0.164–0.450) and 0.171 (95% CI: 0.088–0.255), respectively. The longer the smartphone use time and total screen time, the higher the risk of abnormal academic stress, with OR values of 1.199 (95% CI: 1.103–1.303) and 1.104 (95% CI: 1.056–1.154), respectively. After stratifying by grade group, positive associations between smartphone use time or total screen time and abnormal academic stress were observed in primary and middle school students; for high school students; however, only smartphone use time had a positive association.

**Conclusions:**

This study confirmed that the academic stress is widespread among students aged 10–19 in Shanghai, China. From a public health perspective, smartphone use time and total screen time should therefore be restricted for reducing academic stress and preventing related problems among adolescents in Shanghai, China, in school, family, and other environments.

## Introduction

Learning is considered the most important task during the period of adolescence. Accordingly, adolescents often identify academic stress as their most significant source of stress (1, 2). Many studies have reported physical and psychological problems related to excessive academic stress (3, 4). Buzek et al. found positive associations between academic stress and insufficient sleep or being overweight (5). Zhu et al. found that academic stress was negatively associated with physical activity and sleep but positively associated with anxiety and depression among Chinese adolescents (6). Another study found that the risk of depression for students with academic stress was 2.4 times higher than that of students without academic stress (7). Several studies have also shown that excessive academic stress can lead to anxiety, depression, and suicide (8–11).

Academic stress is a widespread phenomenon observed at different educational stages. A study in the Czech Republic and Poland found that 29.5% of average 16-year-old students experienced abnormal academic stress (3). A study in Nepal observed a prevalence of academic stress among 26.5% of high school students (12). Jayanthi et al. (7) found that more than 80% of adolescents in India students experienced mild academic stress. Meanwhile, many studies have suggested that Chinese adolescents experience more academic stress than those in other countries, such as the US, Japan, and Korea (13–15). Liu et al. found that Chinese high school students had relatively high levels of academic stress, with no significant differences observed between girls and boys (13). Chinese students have also been found to spend more time on classes and homework than those in Western countries (16).

With advancements in mobile and Internet technologies, smartphones, and other devices have become increasingly prevalent, offering functions such as games, videos, and messaging and the ability to process large amounts of information (17). Given the high accessibility of such devices, primary and middle school students now have more opportunities to use them. A study in US found a smartphone use rate of 89% among those aged 13–17 (18). Smartphone use among Chinese adolescents rose from 27.2% in 2011 to >70% in 2017 (19). A study in Hong Kong reported that 98.9%, 56.3%, and 50.9% of students had a computer, TV gaming console, or mobile gaming device at home, respectively, and spent 21.96 h of week on computer-related activities (20). Meanwhile, a 2019 study in Spain found that screen time among adolescents averaged three and a half hours a day (21).

Teachers increasingly use smartphones and other electronic devices to arrange, collect and check homework (22). McDonald et al. (23) found that the most common reason for using smartphones and computers among children was to do homework. To some degree, therefore, smartphone or device use time might represent homework quantity, which could increase academic stress. Other primary reasons for smartphone/device use among adolescents include social activity and entertainment (23, 24). One study found that one in three children used smartphones and other devices for social purposes (23). Spending a lot of time using electronic devices for social activity or entertainment may take away from study time and might therefore increase academic stress.

In light of the above, this study hypothesized that associations exist between academic stress and smartphone use time or total screen time. We therefore used a cohort study design to investigate smartphone use time and total screen time among students aged 10–19 in Shanghai, China, and examine their effects on academic stress.

## Materials and Methods

### Study Design

**Figure 1:**
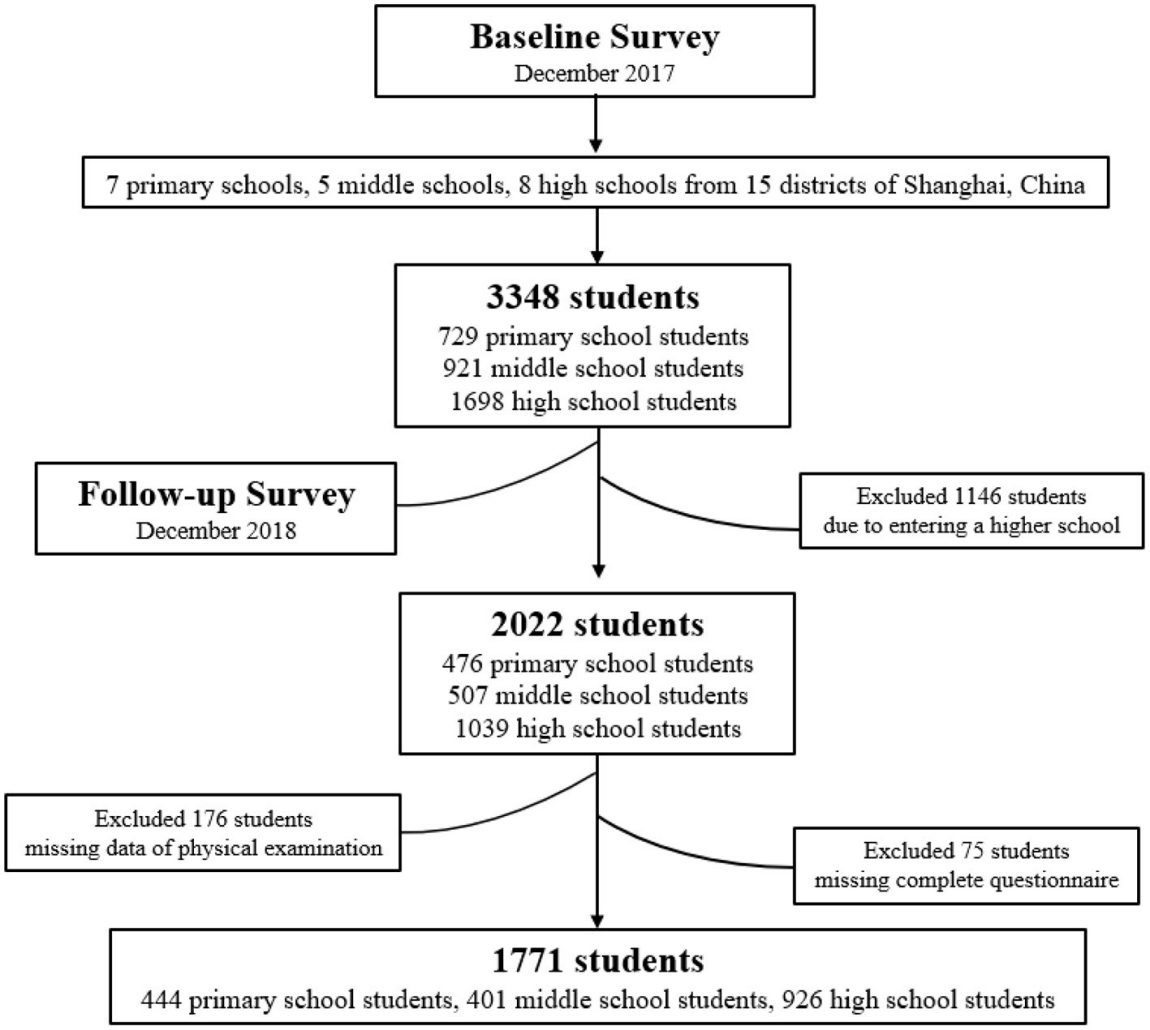
The flowchart of this cohort study.

This research adopted a cohort study design. Baseline and follow-up investigations were conducted in December 2017 and December 2018, respectively. Figure 1 presents a flowchart of the study. In the baseline survey, two-stage nonprobability sampling was used to recruit participants. In the first stage, 20 schools were selected from 15 districts in Shanghai, China, including seven primary schools, five middle schools, and eight high schools. The criteria of school selection were including (1) the principle of voluntary participation; (2) the available physical examination data; (3) the extensive distributions from 15 districts of Shanghai, China; (4) the half of schools from suburb area, and the half of schools from urban area. In the second stage, at least two classes were selected from each school, and all students in the selected classes were enrolled in the study. Follow-up survey was conducted after 1 year for participants in the baseline survey. The follow-up survey method was consistent with that used for the baseline survey. The surveys were conducted in accordance with the Declaration of Helsinki and were approved by the Ethics Committee of Medical Research, School of Public Health, Fudan University (Project identification code: IRB#2018-12-0723; data: 7 December 2018).

### Study Participants

A total of 3,348 students participated in the baseline survey, including 729 primary school students, 921 middle school students, and 1,698 high school students. A total of 1,146 participants were lost in the follow-up survey because they had entered a higher-grade group. Thus, 2,022 students completed the follow-up survey, including 476 primary school students, 507 middle school students, and 1,039 high school students. We excluded 176 students because of missing physical examination data, and 75 students were excluded because of incomplete questionnaires. Finally, 1,771 students aged 10–19 were retained, including 444 primary school students, 401 middle school students, and 926 high school students. Supplementary Table 1 compared the characteristics of excluded and retained participants. Supplementary Table 2 compared the screen time between excluded participants and remained participants in baseline survey by different grade groups. There was no significant difference between the excluded and retained participants, excepted for age, grade group, and smartphone use time and total screen time of middle school students.

### Questionnaire Survey and Physical Examinations

The questionnaire survey was conducted by trained investigators. The questionnaire had several parts, covering demographic information (e.g., age, sex, grade group, birth place) and lifestyle behaviors (physical activity time, sedentary time, smartphone use time, total screen time). The variable that physical activity time was evaluated by asking the question “How long did you do indoor/outdoor physical activities per day in the recent school term? (including running, swimming, playing ball games, et al.”. The variable that sedentary time was evaluated by asking the question “What was your average sedentary time per day? (including class hours at school, homework at home, watching TV or playing games while sitting, et al.)”. The variable that total screen time was evaluated by asking the question “How many hours did you use electronic devices every day? (including smartphone, TV, TV games, computer, tablets, and other electronic devices with access to the Internet, et al.)”. Smartphone use time and total screen time were captured by calculating average smartphone use time and total screen time in the baseline and follow-up surveys. In the follow-up survey, academic stress was evaluated using the Academic Stress Subscale of Mental Health Inventory of Middle School Students (MMHI-60) (25), whose reliability and validity had been verified. Academic Stress Subscale of MMHI-60 was developed by Professor Wang et al. (26) from the Institute of Psychology of the Chinese Academy of Science based on the Symptom Checklist-90 (27), which had been widely used to measure academic stress among Chinese primary and middle school students (28, 29). The scale measures six items: “I feel a heavy study burden,” “I always worry that the teacher will ask me questions in class,” “I always get butterflies in my stomach when I hear about an exam,” “I hate doing homework,” “I hate going to school,” and “I hate taking exams.” Participants rated these statements on a five-point scale: 1 = “not at all,” 2 = “mild,” 3 = “moderate,” 4 = “a little severe,” and 5 = “severe.” An informed consent form was also included in the questionnaire.

Physical examinations were performed by professional school physicians and recorded in the students' medical examination reports. Height and weight were recorded, with accuracies within 0.1 cm and 0.1 kg, respectively. The physical examination data were acquired based on the students' most recent medical examination reports. Body Mass Index (BMI, kg/m^2^) was calculated based on the formula BMI = weight (kg)/height^2^ (m^2^) (30).

### Quality Control

Some quality-control measures were used to ensure the smooth execution of the surveys. First, the investigators were trained to ensure survey quality. Second, to ensure that participants understood the questionnaire content, the investigators could answer any questions they might have, but without directing their responses. Third, the collected questionnaires were double inputted and validated.

### Statistical Analysis

Academic stress score was calculated by integrating the scores for the six items of the Academic Stress Subscale of MMHI-60. Based on the recommendation of Yue et al., academic stress grading was classified as normal (average academic stress score < 2), mild (2 ≤ average academic stress score <3), moderate (3 ≤ average academic stress score < 4), or severe (4 ≤ average academic stress score < 5) (28). Continuous variables were described as mean ± standard deviation (SD). Categorical variables were described as frequencies (ratios). Categorical variables were expressed as gender (male, female), grade group (primary school, middle school, high school), physical activity time (<60 mins/day, ≥ 60 mins/day), sedentary time (< 4 h/day, ≥ 4 h/day), academic stress grading (normal, mild, moderate, severe), and birth place (native place, outside place).

Chi-squared tests or *t*-tests were used for the equilibrium test between smartphone use time, total screen time, age, BMI, gender, academic stress score, academic stress grading, total physical activity, sedentary time, birth place, and grade group. Variance analysis was used to compare differences between smartphone use time and total screen time for different academic stress gradings. A mild, moderate, or severe academic stress grading was classified as abnormal academic stress for the logistic regression analysis. Smartphone use time, total screen time, age, BMI, gender, total physical activity, sedentary time, birth place, and grade group were used as independent variables, and the academic stress score or abnormal academic stress as the dependent variable. The multiple linear regression was used to explore the associations between smartphone use time or total screen time and academic stress score. The multivariate logistic regression model and logistic model stratified by grade group were used to explore the potential associations between abnormal academic stress and smartphone use time or total screen time by calculating the odds ratios (ORs) and corresponding 95% confidence intervals (95% CI). All statistical analyses were conducted using SAS software version 9.2 (SAS Institute Inc., Cary, NC, USA), and two-sided *P* < 0.05 were considered statistically significant.

## Results

Table 1 shows the general characteristics of the participants stratified by grade groups. In 1,771 students participating in the baseline survey and follow-up survey, the average smartphone use times were 1.63 and 1.86 h/day, and the average total screen times were 2.73 and 3.45 h/day, with a gradual increasing trend, respectively. Of the 1,771 students aged 10–19, 444 (25.07%) were primary school students, 401 (22.64%) were middle school students, and 926 (52.29%) were high school students. The average smartphone use times for primary, middle, and high school students were 0.76 ± 0.90, 1.34 ± 1.29, and 2.39 ± 1.66 h/day, respectively; total screen time for the three groups was 2.60 ± 2.63, 2.65 ± 3.39, and 3.52 ± 2.7 h/day (*P* < 0.001). The academic stress scores for primary, middle, and high school students were 9.25 ± 3.96, 11.97 ± 4.58, 15.06 ± 5.10 (out of 30), respectively. The proportions of primary, middle, and high school students in the mild academic stress group were 50 (11.26%), 133 (33.17%), and 405 (43.74%), respectively; 28 (6.31%), 38 (9.48%), and 226 (24.41%) were in the moderate academic stress group, and 2 (0.45%), 11 (2.74%), and 65 (7.02%) were in the severe academic stress group (*P* < 0.001).

**Table 1:** The general characteristics of study population stratified by grade group (*n* = 1,771).

**Characteristics**	**Categories**	**Primary school (*n* = 444)**	**Middle school (*n* = 401)**	**High school (*n* = 926)**	***P*** **value**
Age (years), Mean ± SD		10.38 ± 0.50	14.03 ± 0.75	17.20 ± 0.72	<0.001
Smartphone use time (hour/day), Mean ± SD		0.76 ± 0.90	1.34 ± 1.29	2.39 ± 1.66	<0.001
Total screen time (hour/day), Mean ± SD		2.60 ± 2.63	2.65 ± 3.39	3.52 ± 2.71	<0.001
BMI (kg/m^2^), Mean ± SD		18.21 ± 3.80	19.97 ± 3.56	22.15 ± 4.37	<0.001
Academic stress score (points), Mean ± SD		9.25 ± 3.96	11.97 ± 4.58	15.06 ± 5.10	<0.001
Gender, n (%)	Male	215 (48.42)	216 (53.87)	432 (46.65)	0.054
	Female	229 (51.58)	185 (46.13)	494 (53.35)	
Birth place, n (%)	Native place	325 (73.20)	272 (67.83)	879 (94.92)	<0.001
	Outside place	119 (26.80)	129 (32.17)	47 (5.08)	
Academic stress grading, n (%)	Normal	364 (81.98)	219 (54.61)	230 (24.84)	<0.001
	Mild	50 (11.26)	133 (33.17)	405 (43.74)	
	Moderate	28 (6.31)	38 (9.48)	226 (24.41)	
	Severe	2 (0.45)	11 (2.74)	65 (7.02)	
Physical activity time (min/day), n (%)	<60 mins/day	323 (72.75)	300 (74.81)	740 (79.91)	<0.001
	≥60 mins/day	121 (27.25)	101 (25.19)	186 (20.09)	
Sedentary time (hour/day), n (%)	<4 h/day	286 (64.41)	143 (35.66)	149 (16.09)	<0.001
	≥4 h/day	158 (35.59)	258 (64.34)	777 (83.91)	

*BMI, Body Mass Index; SD, standard deviation*.

**Figure 2:**
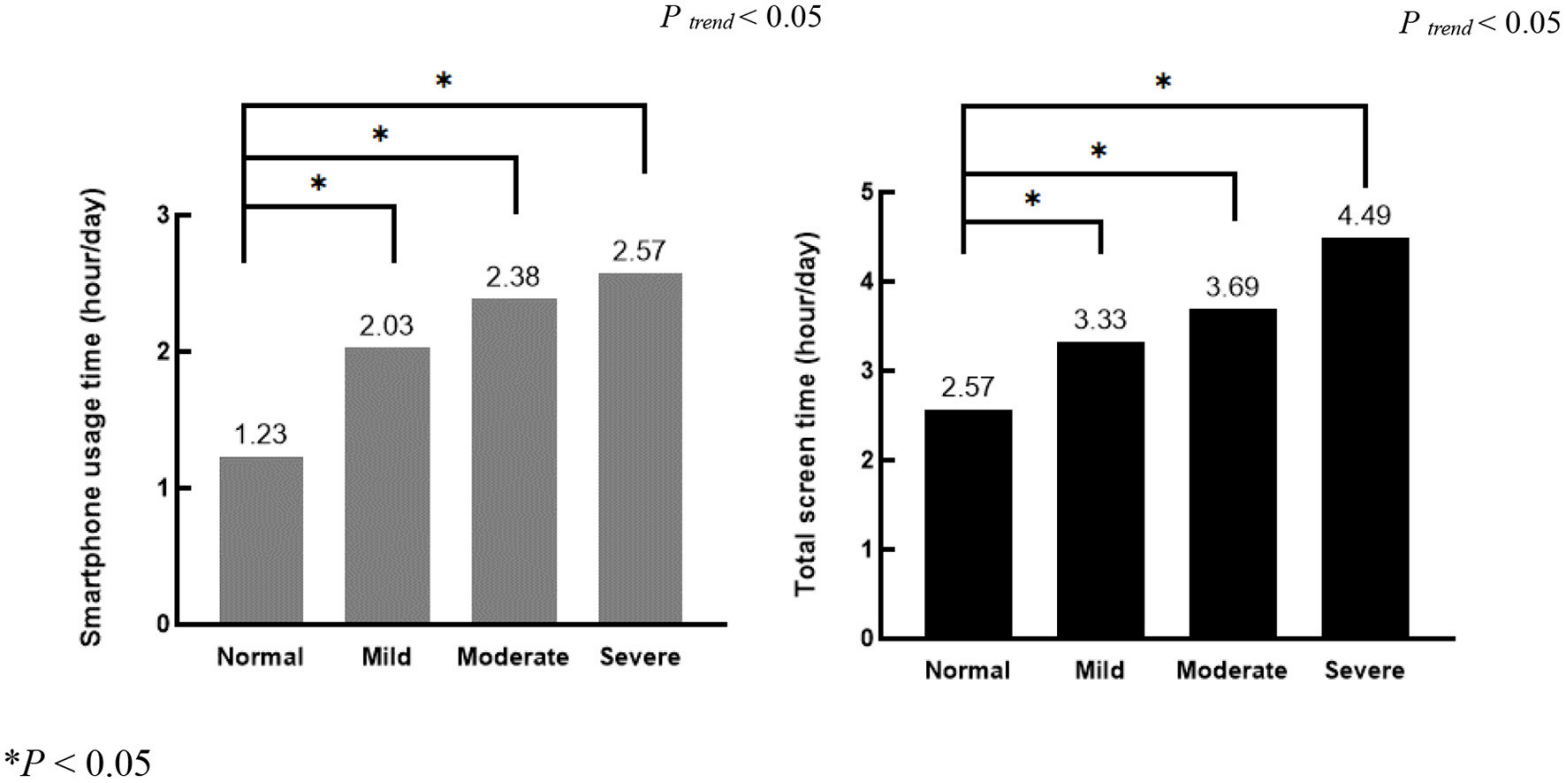
Variance analysis for smartphone use time and total screen time in the different academic stress gradings.

Figure 2 shows the differences in smartphone use time and total screen time between the normal group and the mild, moderate, and severe academic stress groups. Compared with the normal group, smartphone use time and total screen time were higher in the mild, moderate, and severe academic stress groups (*P* < 0.05). Meanwhile, smartphone use time and total screen time showed an increasing trend with the severity of academic stress gradings (*P*_*trend*_ < 0.05).

Table 2 found the correlations of smartphone use time or total screen time with academic stress score. After adjusting for covariables, smartphone use time and total screen time were positively associated with academic stress score in students, with the β values of 0.307 (95% CI: 0.164–0.450, *P* < 0.001) and 0.171 (95% CI: 0.088–0.255, *P* < 0.001), respectively. Meanwhile, two models indicated that age was positively correlated with academic stress score, with the β values of 0.413 (95% CI: 0.096–0.731, *P* = 0.011) and 0.420 (95% CI: 0.103–0.738, *P* = 0.010). In addition, middle school student had higher academic stress score compared with primary school student, with the β values of 2.301 (95% CI: 0.553–4.049, *P* = 0.010) and 2.358 (95% CI: 0.605–4.110, *P* = 0.008) in two models.

**Table 2:** Associations of smartphone use time or total screen time with academic stress score by multiple linear regression.

**Characteristics**	**Categories**	**Model 1^a^**		**Model 2^b^**	
		**β (95% CI)**	***P*** **value**	**β (95% CI)**	***P*** **value**
Smartphone use time		0.307 (0.164–0.450)	<0.001	–	–
Total screen time		–	–	0.171 (0.088–0.255)	<0.001
Age		0.413 (0.096–0.731)	0.011	0.420 (0.103–0.738)	0.010
BMI		0.035 (−0.019–0.09)	0.201	0.036 (-0.018–0.09)	0.194
Gender	Male	1 (reference)		1 (reference)	
	Female	−0.115 (−0.555–0.326)	0.609	−0.022 (−0.464–0.419)	0.921
Grade group	Primary school	1 (reference)		1 (reference)	
	Middle school	2.301 (0.553–4.049)	0.010	2.358 (0.605–4.110)	0.008
	High school	1.232 (−1.445–3.908)	0.367	1.480 (−1.185–4.144)	0.276
Birth place	Native place	1 (reference)		1 (reference)	
	Outside place	0.331 (−0.287–0.949)	0.294	0.315 (−0.304–0.934)	0.318
Physical activity time (min/day)	<60	1 (reference)		1 (reference)	
	≥60	−0.030 (−0.550–0.490)	0.910	0.005 (−0.515–0.525)	0.986
Sedentary time (hour/day)	<4	1 (reference)		1 (reference)	
	≥4	−0.327 (−0.845–0.190)	0.215	−0.311 (−0.828–0.206)	0.239

*BMI, Body Mass Index; CI, credibility interval*.

a *The model 1 was adjusted for smartphone use time, age, BMI, gender, grade group, birth place, physical activity time and sedentary time*;

a *The model 2 was adjusted for total screen time, age, BMI, gender, grade group, birth place, physical activity time and sedentary time*.

Table 3 shows the associations between smartphone use time or total screen time and abnormal academic stress. Logistic regression revealed that the higher the smartphone use time and total screen time, the higher the risk of abnormal academic stress, with OR values of 1.199 (95% CI: 1.103–1.303) and 1.104 (95% CI: 1.056–1.154), respectively. There was a higher risk of abnormal academic stress among participants with increased age in the two models, resulting in OR values of 1.179 (95% CI: 1.011–1.376) and 1.193 (95% CI: 1.022–1.392). Moreover, middle school students had a higher risk of abnormal academic stress than primary school students in the two models, with OR values of 2.601 (95% CI: 1.041–6.497) and 2.939 (95% CI: 1.174–7.359).

**Table 3:** Associations between smartphone use time or total screen time and abnormal academic stress by multivariable logistic regression.

**Characteristics**	**Categories**	**Model 1^a^**		**Model 2^b^**	
		**OR (95% CI)**	***P*** **value**	**OR (95% CI)**	***P*** **value**
Smartphone use time		1.199 (1.103–1.303)	<0.001	–	–
Total screen time		–	–	1.104 (1.056–1.154)	<0.001
Age		1.179 (1.011–1.376)	0.036	1.193 (1.022–1.392)	0.026
BMI		1.015 (0.987–1.043)	0.292	1.016 (0.989–1.044)	0.242
Gender	Male	1 (reference)		1 (reference)	
	Female	1.002 (0.806–1.246)	0.985	1.054 (0.847–1.311)	0.639
Grade Group	Primary school	1 (reference)		1 (reference)	
	Middle school	2.601 (1.041–6.497)	0.041	2.939 (1.174–7.359)	0.021
	High school	2.876 (0.751–11.006)	0.123	3.231 (0.847–12.316)	0.086
Birth place	Native place	1 (reference)		1 (reference)	
	Outside place	1.095 (0.809–1.483)	0.555	1.082 (0.799–1.465)	0.610
Physical activity time (min/day)	<60	1 (reference)		1 (reference)	
	≥ 60	0.901 (0.697–1.166)	0.429	0.908 (0.702–1.176)	0.465
Sedentary time (hour/day)	<4	1 (reference)		1 (reference)	
	≥ 4	0.889 (0.689–1.149)	0.370	0.895 (0.692–1.156)	0.396

*BMI, Body Mass Index; OR, odd ratio; CI, credibility interval*.

a *The model 1 was adjusted for smartphone use time, age, BMI, gender, grade group, birth place, physical activity time and sedentary time*;

b *The model 2 was adjusted for total screen time, age, BMI, gender, grade group, birth place, physical activity time and sedentary time*.

Tables 4, 5 show the associations between abnormal academic stress and smartphone use time or total screen time stratified by grade group. After adjusting for covariables, smartphone use time and total screen time were positively associated with abnormal academic stress, with OR values of 1.466 (95% CI: 1.147–1.874) and 1.181 (95% CI: 1.085–1.286) for primary school students and 1.284 (95% CI: 1.063–1.550) and 1.100 (95% CI: 1.003–1.206) for middle school students. For high school students, the significantly positive association was only found for smartphone use time, with an OR value of 1.129 (95% CI: 1.022–1.247). However, there was a marginal positive association between total screen time and abnormal academic stress in high school students, with an OR value of 1.061 (95% CI: 0.997–1.128). In addition, the two models showed that age was positively associated with abnormal academic stress only for high school students, with OR values of 1.239 (95% CI: 1.005–1.528) and 1.247 (95% CI: 1.012–1.538).

**Table 4:** Associations between smartphone use time and the abnormal academic stress stratified by grade group.

**Characteristics**	**Categories**	**Primary school**	**Middle school**	**High school**
		**OR (95% CI)**	**OR (95% CI)**	**OR (95% CI)**
Smartphone use time		1.466 (1.147–1.874)	1.284 (1.063–1.550)	1.129 (1.022–1.247)
Age		1.038 (0.634–1.701)	1.067 (0.813–1.399)	1.239 (1.005–1.528)
BMI		0.972 (0.905–1.043)	1.063 (1.003–1.127)	1.014 (0.978–1.051)
Gender	Male	1 (reference)	1 (reference)	1 (reference)
	Female	1.093 (0.660–1.809)	0.692 (0.460–1.041)	1.204 (0.885–1.638)
Birth place	Native place	1 (reference)	1 (reference)	1 (reference)
	Outside place	1.162 (0.671–2.012)	1.023 (0.636–1.647)	0.941 (0.476–1.860)
Physical activity time	<60	1 (reference)	1 (reference)	1 (reference)
(min/day)	≥ 60	0.575 (0.307–1.074)	0.833 (0.513–1.350)	1.180 (0.803–1.735)
Sedentary time (hour/day)	<4	1 (reference)	1 (reference)	1 (reference)
	≥ 4	1.031 (0.609–1.745)	0.845 (0.549–1.301)	0.900 (0.595–1.361)

*BMI, Body Mass Index; OR, odd ratio; CI, credibility interval*.

*The model was adjusted for smartphone use time, age, BMI, gender, birth place, physical activity time and sedentary time stratified by grade group*.

**Table 5:** Associations between total screen time and the abnormal academic stress stratified by grade group.

**Characteristics**	**Categories**	**Primary school**	**Middle school**	**High school**
		**OR (95% CI)**	**OR (95% CI)**	**OR (95% CI)**
Total screen time		1.181 (1.085–1.286)	1.100 (1.003–1.206)	1.061 (0.997–1.128)
Age		1.043 (0.634–1.714)	1.089 (0.832–1.427)	1.247 (1.012–1.538)
BMI		0.967 (0.900–1.039)	1.066 (1.006–1.129)	1.016 (0.980–1.053)
Gender	Male	1 (reference)	1 (reference)	1 (reference)
	Female	1.140 (0.685–1.897)	0.725 (0.481–1.093)	1.251 (0.920–1.701)
Birth place	Native place	1 (reference)	1 (reference)	1 (reference)
	Outside place	1.074 (0.614–1.881)	1.065 (0.662–1.716)	0.969 (0.492–1.910)
Physical activity time	<60	1 (reference)	1 (reference)	1 (reference)
(min/day)	≥ 60	0.616 (0.330–1.150)	0.829 (0.513–1.342)	1.175 (0.800–1.727)
Sedentary time (hour/day)	<4	1 (reference)	1 (reference)	1 (reference)
	≥ 4	1.028 (0.604–1.748)	0.835 (0.543–1.282)	0.914 (0.604–1.381)

*BMI, Body Mass Index; OR, odd ratio; CI, credibility interval*.

*The model was adjusted for total screen time, age, BMI, gender, birth place, physical activity time and sedentary time stratified by grade group*.

## Discussion

To our knowledge, this cohort study is the first to assess smartphone use time and total screen time among students aged 10–19 in Shanghai, China, and examine the effects on academic stress. Existing studies have mainly reported academic stress among college students (7, 31, 32) with few focusing on adolescents. Our findings indicate that the academic stress of students aged 10–19 should not be ignored, even among primary school students. Variance analysis showed that smartphone use time and total screen time were significantly higher in the mild, moderate, and severe academic stress groups than in the normal group, showing an increasing trend with academic stress severity. Linear and logistic regression indicated that the higher the smartphone use time and total screen time, the higher the academic stress score, the higher the risk of abnormal academic stress.

Compared with other countries, Chinese adolescents might experience more academic stress from a young age. We found that the proportions of abnormal academic stress were 18.02, 45.39, and 75.16% for primary, middle, and high school students, respectively. Meanwhile, the proportions of moderate and severe academic stress were 6.76, 12.22, and 31.43% for primary, middle, and high school students, respectively. However, a study in the Czech Republic and Poland revealed that only an average of 29.50% of students had academic stress (3). That result was consistent with a study in Nepal, which found that 26.5% of high school students experienced academic stress (12). This phenomenon may be related to different educational systems between China and other countries (33). Young Chinese students tend to face severe academic competition, as with the notoriously difficult college entrance examinations (34). Students and their parents often prepare intensively from early childhood for such competition, including participation in various extracurricular tutorial classes. One study found that more than 62% of primary school students in China attended extracurricular tutorial classes (35), mostly aiming to improve subject knowledge (36). This has caused Chinese students to have more academic stress than those from other countries (37–39).

Academic stress was found to increase with increased age and grade from 18.02% to 75.16%. This is partially consistent with a study in Chongqing, China, that found proportions of abnormal academic stress of 19.9, 55.0, and 62.6% among primary, middle, and high school students, respectively (28). As students move into higher grades, increased subject difficulty and number might lead to more academic stress. Therefore, particular attention should be paid to academic stress among students in the upper grades.

Our results showed that the average duration of smartphone use time and total screen time was about 2–5 h/day for the participants. Haug et al., meanwhile, found that smartphone use time was 1–2 h/day among 33.0% of reporting young people in Switzerland (40). This is consistent with another Chinese study in which 38.2% of students reported 2–4 h/day of smartphone use (41). Meanwhile, we found that there was an increasing trend for smartphone use time and total screen time in students with the increase of age. In our cohort study, the results confirmed that the smartphone use time and total screen time were positively associated with the academic stress score, and increased the risk of abnormal academic stress. One reason could be that smartphones and other devices are used for homework. This is supported by a study (23), which found that completing homework was the main reason why children used smartphones and other devices. Thus, device use time might to some extent represent the amount of homework students' must complete and their associated academic stress. Another reason could be related to using electronic devices for social activity and entertainment (42). One study found that more than 30% of adolescents used smartphones and other devices for social activity (23). Devoting a lot of time to social activity and entertainment could take away from study time, thus adversely affecting students' study progress and increasing their academic stress. Meanwhile, due to backward study progress, increasing academic stress might be further increased the screen time, and they were in a bidirectional relationship. Moreover, using smartphone to social activity and entertainment may prevent students from focusing on their study, which may lead to the poor academic performance. Kwok et al. found the significantly negative association between smartphone addiction and academic performance (43). The poor academic performance may cause the further increase of academic stress. Therefore, to reduce academic stress, parents should seek to restrict smartphone use related to social activity and entertainment. Teachers, meanwhile, should reduce the amount of homework assigned using smartphones and other devices.

Another interesting finding was that the positive association between academic stress and smartphone use time or total screen time were found for primary and middle school students. For high school students, however, this positive association existed only for smartphone use time, not total screen time. There was a marginal positive association between total screen time and academic stress in high school students. This result showed that high school students may spend most of their screen time on their mobile phones (44). Meanwhile, we found the OR values of smartphone use time and total screen time were decreasing with the increase of grade group, which revealed that the influence degree of smartphone use time and total screen time on academic stress decreased with the increase in grade group. Since parents' and schools' academic expectations increase with grade level, academic stress is pervasive among high school students (8, 45). Thus, high school students' academic stress was not readily influenced by individual behaviors, such as the use of smartphones and other devices. The situation was different for primary and middle school students, whose individual behaviors affected their level of academic stress. In short, it is important to encourage the appropriate use of electronic devices from an early age.

## Strengths and Limitations

The present study has several strengths. The cohort study design was adopted in this study, and the sample size was relatively large. The study participants were including primary, middle and high school student from the 15 districts of Shanghai, China, and had the better representation. However, some limitations also exist in our study. First, we were unable to distinguish between using electronic devices for homework and for entertainment, which need to be identified in future studies. Second, the cohort study only used data at two time points with an interval of one year. Due to the academic stress only investigated in the follow-up survey, we can't observe the change trend of the academic stress in study participants. Third, some other factors that might affect academic stress were not included, such as sleep duration, neurodevelopmental disorders and diseases, parental expectation, peer stress, and interpersonal communication (7, 8, 43, 46). These factors can be considered in future research. Last, because there was a significant difference of smartphone use time and total screen time between excluded and retained participants in middle school students, which may affect the results of this study for middle school students.

## Conclusion

This cohort study found that academic stress was widespread among students aged 10–19 in Shanghai, China—even among primary school students. Meanwhile, smartphone use time and total screen time were identified as potential reasons for increased academic stress among the participants. From a public health perspective, smartphone use time and total screen time should be restricted for reducing academic stress and preventing various related problems among students aged 10–19 in Shanghai, China, in school, family, and other environments.

## Data Availability Statement

The raw data supporting the conclusions of this article will be made available by the authors, without undue reservation.

## Ethics Statement

The studies involving human participants were reviewed and approved by Ethics Committee of Medical Research, School of Public Health, Fudan University. Written informed consent to participate in this study was provided by the participants' legal guardian/next of kin.

## Author Contributions

SL and YJ designed the research, wrote the paper, and had primary responsibility for the final content. YL provided the data. SL performed the statistical analyses. YJ, GH, and BC reviewed and edited the manuscript. All authors have read and agreed to the published version of the manuscript.

## Funding

This research was funded by the Shanghai Municipal Health Commission, grant numbers GWV-10.2-YQ23 and GWV-10.1-XK14.

## Conflict of Interest

The authors declare that the research was conducted in the absence of any commercial or financial relationships that could be construed as a potential conflict of interest.

## Publisher's Note

All claims expressed in this article are solely those of the authors and do not necessarily represent those of their affiliated organizations, or those of the publisher, the editors and the reviewers. Any product that may be evaluated in this article, or claim that may be made by its manufacturer, is not guaranteed or endorsed by the publisher.

## Abbreviations

MMHI-60: Mental Health Inventory of Middle School Students
ORs: odds ratios
BMI: Body Mass Index
SD: standard deviation
CI: credibility interval.

## References

[1] Anda Dd Baroni S Boskin L Buchwald L Morgan J Ow J. Stress, stressors and coping among high school students. Child Youth Serv Rev. (2000) 22:441–63. 10.1016/S0190-7409(00)00096-7

[2] Kouzma NM Kennedy GA. Self-reported sources of stress in senior high school students. Psychol Rep. (2004) 94:314–6. 10.2466/pr0.94.1.314-316 15077784

[3] Fromel K Safar M Jakubec L Groffik D Zatka R. Academic stress and physical activity in adolescents. Biomed Res Int. (2020) 2020:4696592. 10.1155/2020/4696592 32185205 PMC7060887

[4] Deb S Strodl E Sun J. Academic stress, parental pressure, anxiety and mental health among indian high school students. Int J Psyco Behav Sci. (2015) 5:26–34. 10.5923/j.ijpbs.20150501.04

[5] Buzek T Poulain T Vogel M Engel C Bussler S Korner A. Relations between sleep duration with overweight and academic stress-just a matter of the socioeconomic status? Sleep Health. (2019) 5:208–15. 10.1016/j.sleh.2018.12.004 30928123

[6] Zhu X Haegele JA Liu H Yu F. Academic stress, physical activity, sleep, and mental health among chinese adolescents. Int J Environ Res Public Health. (2021) 18:7257. 10.3390/ijerph18147257 34299708 PMC8304898

[7] Jayanthi P Thirunavukarasu M Rajkumar R. Academic stress and depression among adolescents: a cross-sectional study. Indian Pediatr. (2015) 52:217–9. 10.1007/s13312-015-0609-y 25848997

[8] Ali NM Nowshad NA Mansoor KM Ibnouf RA Albehiery RM Carrick FR. Perceived Academic and Psychological Stress among Adolescents in United Arab Emirates: Role of gender, age, dep ression, and high expectation of parents. Psychiatr Danub. (2019) 31:331–7. 31488749

[9] Romo-Nava F Tafoya SA Gutierrez-Soriano J Osorio Y Carriedo P Ocampo B. The association between chronotype and perceived academic stress to depression in medical students. Chronobiol Int. (2016) 33:1359–68. 10.1080/07420528.2016.1217230 27579890

[10] Lemay V Hoolahan J Buchanan A. Impact of a yoga and meditation intervention on students' stress and anxiety levels. Am J Pharm Educ. (2019) 83:7001. 10.5688/ajpe7001 31333265 PMC6630857

[11] Wang X Hegde S Son C Keller B Smith A Sasangohar F. Investigating mental health of US college students during the COVID-19 pandemic: cross-sectional survey study. J Med Internet Res. (2020) 22:e22817. 10.2196/22817 32897868 PMC7505693

[12] Gurung M Chansatitporn N Chamroonsawasdi K Lapvongwatana P. Academic stress among high school students in a rural area of nepal: a descriptive cross-sectional study. JNMA J Nepal Med Assoc. (2020) 58:306–9. 10.31729/jnma.4978 32538923 PMC7654462

[13] Liu Y Lu Z. Chinese high school students' academic stress and depressive symptoms: gender and school climate as moderators. Stress Health. (2012) 28:340–6. 10.1002/smi.2418 22190389

[14] Sun JD Dunne MP Hou XY Xu AQ. Educational stress among Chinese adolescents: individual, family, school and peer influences. Educ Rev. (2013) 65:284–302. 10.1080/00131911.2012.659657

[15] Chen X Glaude M. Academic stress among Chinese adolescents: can psychological stress theory explain this tragedy. Int J Humanit Soc Sci. (2017) 7:17–25.

[16] Chen CF Yang N Sages J. A. comparative investigation on the learning stress and extracurricular activities between senior high school students in China and Sweden. J Huzhou Univ. (2014) 36:97–102. 10.3969/j.issn.1009-1734.2014.12.021

[17] Miralles I Granell C Díaz-Sanahuja L Van Woensel W Bretón-López J Mira A. Smartphone apps for the treatment of mental disorders: systematic review. JMIR Mhealth Uhealth. (2020) 8:e14897. 10.2196/14897 32238332 PMC7163422

[18] Abi-Jaoude E Naylor KT Pignatiello A. Smartphones, social media use and youth mental health. CMAJ. (2020) 192:E136–41. 10.1503/cmaj.190434 32041697 PMC7012622

[19] Ji WM Shen J Yang BY Ji L. Annual report on the Internet use and reading practice of Chinese minors (2017-2018). Soc Sci Acad Press (CHINA) Beijing. (2018).

[20] Siu DC Tse LA Yu IT Griffiths SM. Computer products usage and prevalence of computer related musculoskeletal discomfort among adolescents. Work. (2009) 34:449–54. 10.3233/WOR-2009-0945 20075522

[21] Cabre-Riera A Torrent M Donaire-Gonzalez D Vrijheid M Cardis E Guxens M. Telecommunication devices use, screen time and sleep in adolescents. Environ Res. (2019) 171:341–7. 10.1016/j.envres.2018.10.036 30716511

[22] Chen HL. Design and implementation of an electronic assignment system based on WeChat mini program. J Guiyang Univ (Nat Sci). (2021) 16:88-91+97. 10.16856/j.cnki.52-1142/n.2021.02.018

[23] McDonald JA Sroka C Olivares E Marin M Gurrola M Sharkey JR. Patterns of screen time among rural Mexican-American children on the New Mexico-Mexico border. Prev Chronic Dis. (2018) 15:E113. 10.5888/pcd15.180070 30218553 PMC6157263

[24] Meng H Cao H Hao R Zhou N Liang Y Wu L. Smartphone use motivation and problematic smartphone use in a national representative sample of Chinese adolescents: The mediating roles of smartphone use time for various activities. J Behav Addict. (2020) 9:163–74. 10.1556/2006.2020.00004 32359238 PMC8935195

[25] Wu Z Liu Z Zou Z Wang F Zhu M Zhang W. Changes of psychotic-like experiences and their association with anxiety/depression among young adolescents before COVID-19 and after the lockdown in China. Schizophr Res. (2021) 237:40–6. 10.1016/j.schres.2021.08.020 34481204 PMC8437585

[26] Wang JS Li Y He ES. The preparation and standardization for Mental Health Inventory of 997 Middle School students (MMHI-60) in China. Sci Soc Psychol. (1997) 4:15–20.

[27] Bonicatto S Dew MA Soria JJ Seghezzo ME. Validity and reliability of symptom checklist '90 (SCL90) in an Argentine population sample. Soc Psych Psych Epid. (1997) 32:332–8. 10.1007/BF00805438 9299927

[28] Yue CZ Yang ZW Fei XF Wei C Xiao QG Lin XT. A study on the mental health status and influencing factors of adolescents in a district in Chongqing. Health Med Res Pract. (2021) 18:15–21. 10.11986/j.issn.1673-873X.2021.03.003

[29] Hu Y Han J Chen XG Yang SP Xu YH Xie S. Study on the psychological-health status and its relationship with social capital among left behind children in rural area, Macheng, Hubei province. Chin J Epidemiol. (2014) 35:31–4. 10.3760/cma.j.issn.0254-6450.2014.01.008 24685033

[30] Zhu YB. “Obesity paradox”—A phenomenon based upon the health-related outcome on quality of life. Chin J Epidemiol. (2013) 34:294−6. 10.3760/cma.j.issn.0254-6450.2013.03.020 23759240

[31] McCarthy B Trace A O'Donovan M Brady-Nevin C Murphy M O'Shea M. Nursing and midwifery students' stress and coping during their undergraduate education programmes: an integrative review. Nurse Educ Today. (2018) 61:197–209. 10.1016/j.nedt.2017.11.029 29227889

[32] Huang N Qiu S Alizadeh A Wu H. How incivility and academic stress influence psychological health among college students: the moderating role of gratitude. Int J Environ Res Public Health. (2020) 17:3237. 10.3390/ijerph17093237 32384724 PMC7246712

[33] Wang XH. Compare the differences between Chinese and Western school education systems. J Liaoning Educ Admin Inst. (2005) 11:45–6. 10.3969/j.issn.1672-6022.2005.11.01924871248

[34] Ye L Posada A Liu Y A. Review on the relationship between chinese adolescents' stress and academic achievement. New Dir Child Adolesc Dev. (2019) 2019:81–95. 10.1002/cad.20265 30614631

[35] Wang F. Review and andlysis of the research situation of primary school students participating in extracurricular tutoring classes. J Nanchang Coll Edu. (2017) 32:100–4. 10.3969/j.issn.1008-6757.2017.04.032

[36] Zhong WJ Wang XQ. How to rationally treat extracurricular Remedial Classes—Based on the investigation of the current situation of primary and middle school students attending extracurricular remedial classes in a county of Shandong Province. Theor Pract Contemp Educ. (2017) 9:9–14. 10.13582/j.cnki.1674-5884.2017.03.003

[37] Liu YY Lu ZH. Longitudinal analysis of Chinese high school student's stress in school and academic achievement. Educ Psychol-Uk. (2011) 31:723–9. 10.1080/01443410.2011.600245

[38] Liu YY Lu ZH. The Chinese high school student's stress in the school and academic achievement. Educ Psychol-Uk. (2011) 31:27–35. 10.1080/01443410.2010.513959

[39] Hu X. Comparisons among the daily life of Chinese, Japanese and American middle School Students. Contemp Youth Res. (2001) 4:45−8. 10.3969/j.issn.1006-1789.2001.04.013

[40] Haug S Castro RP Kwon M Filler A Kowatsch T Schaub MP. Smartphone use and smartphone addiction among young people in Switzerland. J Behav Addict. (2015) 4:299–307. 10.1556/2006.4.2015.037 26690625 PMC4712764

[41] Long J Liu TQ Liao YH Qi C He HY Chen SB. Prevalence and correlates of problematic smartphone use in a large random sample of Chinese undergraduates. BMC Psychiatry. (2016) 16:408. 10.1186/s12888-016-1083-3 27855666 PMC5114822

[42] Kurtuncu M Ayyildiz TK Kurt A. An examination of smartphone addiction and loneliness among high school students according to various variables: a sample from Turkey. Perspect Psychiatr Care. (2021) 57:941–7. 10.1111/ppc.12639 33043483

[43] Kwok C Leung PY Poon KY Fung XC. The effects of internet gaming and social media use on physical activity, sleep, quality of life, and academic performance among university students in Hong Kong: a preliminary study. Asian J Soc Health Behav. (2021) 4:36–44. 10.4103/shb.shb_81_20

[44] Sayili U Vehid S Erginoz E. Problematic internet use in turkish high school students: prevalence and related factors. Am J Health Behav. (2021) 45:31–43. 10.5993/AJHB.45.1.3x 33402236

[45] Xu X Xu G Liu M Deng C. Influence of parental academic involvement on the achievement goal orientations of high school students in China: a latent growth model study. Br J Educ Psychol. (2020) 90:700–18. 10.1111/bjep.12326 31680248

[46] Mathew N Khakha DC Qureshi A Sagar R Khakha CC. Stress and coping among adolescents in selected schools in the capital city of India. Indian J Pediatr. (2015) 82:809–16. 10.1007/s12098-015-1710-x 25689960

