# Supplementary material for: Smartphone Use Time and Total Screen Time Among Students Aged 10–19 and the Effects on Academic Stress: A Large Longitudinal Cohort Study in Shanghai, China
Source: Front Public Health. 2022 May 17;10:869218. doi: 10.3389/fpubh.2022.869218 (PMC9152090; doi:10.3389/fpubh.2022.869218)
Supplement: Supplementary file 1 [file Table_1.DOCX]

| **Characteristics** | | **Excluded data (n=1577)** | **Remained data (n=1771)** | ***P* value** |
| --- | --- | --- | --- | --- |
| Age (years), n (%) | ≤15 | 615 (39.00) | 836 (47.20) | < 0.001 |
|  | >15 | 962 (61.00) | 935 (52.80) |  |
| Gender, n (%) | Male | 798 (50.60) | 863 (48.73) | 0.187 |
|  | Female | 779 (49.40) | 908 (51.27) |  |
| Birth place, n (%) | Native place | 1312 (83.20) | 1476 (83.34) | 0.495 |
|  | Outside place | 265 (16.80) | 295 (16.66) |  |
| Grade group, n (%) | Primary school | 278 (17.63) | 444 (25.07) | < 0.001 |
|  | Middle school | 344 (21.81) | 401 (22.64) |  |
|  | High school | 955 (60.56) | 926 (52.29) |  |

**Table S1. The characteristic comparisons between excluded participants and remained participants in baseline survey**

**Table S2. The screen time comparisons between excluded participants and remained participants in baseline survey by different grade groups**

| **Screen time** | **Grade group** | **Excluded data (n=1577)** | **Remained data (n=1771)** | ***P* value** |
| --- | --- | --- | --- | --- |
| Smartphone use time | Primary school students | 0.72 ± 0.83 | 0.80 ± 1.20 | 0.866 |
| (hour/day) | Middle school students | 1.92 ± 1.86 | 1.63 ± 1.64 | < 0.001 |
|  | High school students | 2.15 ± 1.85 | 1.63 ±1.64 | 0.598 |
| Total screen time | Primary school students | 2.49 ± 2.74 | 2.27 ± 2.67 | 0.238 |
| (hour/day) | Middle school students | 3.58 ± 3.47 | 2.73 ± 2.64 | < 0.001 |
|  | High school students | 3.04 ± 2.68 | 2.74 ±2.65 | 0.802 |
